# Hepatocellular carcinoma cell differentiation trajectory predicts immunotherapy, potential therapeutic drugs, and prognosis of patients

**DOI:** 10.1515/biol-2022-0656

**Published:** 2023-08-08

**Authors:** Jun Qiu, Haoyun Wang, Xin Lv, Lipeng Mao, Junyan Huang, Tao Hao, Junliang Li, Shuo Qi, Guodong Chen, Haiping Jiang

**Affiliations:** Department of General Surgery, The First Affiliated Hospital of Jinan University, Guangzhou 510630, Guangdong Province, China; Department of Microbiology and Immunology, Institute of Geriatric Immunology, School of Medicine, Jinan University, Guangzhou 510630, Guangdong Province, China; Department of Clinical Nutrition, The First Affiliated Hospital of Jinan University, Guangzhou 510630, Guangdong Province, China; Department of Neurosurgery, Guangzhou Women and Children’s Medical Center, Guangzhou 510630, Guangdong Province, China; Department of Hepatopancreatobiliary Surgery, The First Affiliated Hospital of University of South China, Hengyang 421001, Hunan Province, China

**Keywords:** cell differentiation trajectory, hepatocellular carcinoma, immune checkpoint, overall survival, molecular-targeted drugs

## Abstract

The aim of this study is to explore a novel classification and investigate the clinical significance of hepatocellular carcinoma (HCC) cells. We analyzed integrated single-cell RNA sequencing and bulk RNA-seq data obtained from HCC samples. Cell trajectory analysis divided HCC cells into three subgroups with different differentiation states: state 1 was closely related to phosphoric ester hydrolase activity, state 2 was involved in eukaryotic initiation factor 4E binding, translation regulator activity and ribosome, and state 3 was associated with oxidoreductase activity and metabolism. Three molecular classes based on HCC differentiation-related genes (HDRGs) from HCC samples were identified, which revealed immune checkpoint gene expression and overall survival (OS) of HCC patients. Moreover, a prognostic risk scoring (RS) model was generated based on eight HDRGs, and the results showed that the OS of the high-risk group was worse than that of the low-risk group. Further, potential therapeutic drugs were screened out based on eight prognostic RS-HDRGs. This study highlights the importance of HCC cell differentiation in immunotherapy, clinical prognosis, and potential molecular-targeted drugs for HCC patients, and proposes a direction for the development of individualized treatments for HCC.

## 1 Introduction

Primary liver cancer, especially hepatocellular carcinoma (HCC), is one of the most common malignant tumors of the digestive system worldwide [1]. According to the latest data released by the International Agency for Research on Cancer of the World Health Organization, the number of new liver cancer cases worldwide is projected to reach 910,000 in 2020, ranking sixth among malignant tumors. The number of deaths caused by liver cancer is expected to reach 830,000, ranking third among malignant tumors [2]. Although significant progress has been made in HCC treatment (e.g., partial hepatectomy, liver transplantation, local ablation therapy, radiotherapy, hepatic arterial infusion chemotherapy, transarterial chemoembolization, molecular-targeted therapeutics, and immunotherapy), HCC is often diagnosed in the middle or advanced stages due to its insidious onset and aggressive nature [3,4]. As a result, HCC patients often have poor prognoses, with an average 5-year survival rate of 19.6% in America, which can drop to as low as 2.5% in advanced cases [5]. HCC is a malignancy with high heterogeneity, which poses significant challenges for its treatment. Research has revealed that the intratumoral heterogeneity of HCC is mainly due to the existence of cancer stem cells (CSCs). Moreover, different CSC populations might harbor distinct oncogenic driver genes, further complicating the development of targeted therapies. Therefore, understanding the complex interplay between CSCs and HCC is essential to advance our molecular understanding of this disease and develop more effective personalized treatments [6]. Therefore, it is crucial to gain a comprehensive understanding of the intratumoral heterogeneity (ITH) of HCC and more accurately interpret the relationship between ITH and HCC at the molecular level. This knowledge is essential for improving the molecular typing and developing individualized treatments for HCC.

It is widely accepted that the formation of tumors results from the gradual accumulation of genetic variations in cells. Tumor cells containing different combinations of mutations lead to cell phenotypic heterogeneity due to genetic heterogeneity, resulting in the formation of different clonal subgroups with varying responses to treatments and metastatic abilities [7,8,9]. The traditional cytogenetic information research method is to perform high-throughput sequencing on a large number of mixed cells to obtain the average gene expression of a group of cells, disregarding the heterogeneity that exists between cells [10]. With the continuous development of sequencing technology, single-cell RNA sequencing (scRNA-Seq) has emerged as a technology that can better understand the heterogeneity of cells and is now widely used in this field [11]. This technology offers a better understanding of the heterogeneity of tumor cells in HCC, enabling the discovery of new cell subgroups and providing insights into the composition of the tumor microenvironment. A study [6] showed that scRNA-Seq was performed on Human liver cancer cell lines (HuH1 and HuH7) with a liver cancer tissue sample, revealing that liver cancer stem cell populations with different molecular markers exhibit distinct transcriptome profiles and stem cell subpopulations. The differentially expressed genes in liver cancer patients are independently correlated with the prognosis of liver cancer patients, indicating that the diversity of liver cancer stem cell transcriptomes is closely related to the heterogeneity of liver cancer cells and the occurrence and development of liver cancer. This provides new opinions for the prognosis analysis and clinical treatments of liver cancer. However, it is still unclear in which different states of differentiation HCC cells exist, whether the new classification of HCC patients based on cell differentiation trajectories is related to tumor biological behavior, and how it may impact immunotherapy, potential molecular-targeted drugs, and patient survival prediction.

Therefore, we conducted a study using scRNA-Seq to explore the differentiation trajectory of HCC cells and combined this with bulk RNA-Seq data to investigate the potential impact on immunotherapy and patient survival rates, and we screen for the potential molecular-targeted drugs. Our study provides a foundation for achieving clinically accurate molecular typing of HCC and offers guidance for individualized treatment plans based on molecular typing.

## 2 Materials and methods

### 2.1 Data acquisition

The scRNA-seq data and bulk RNA-seq data of human HCC samples were obtained from the following three databases in this study: Gene Expression Omnibus (GEO, GSE125449, http://www.ncbi.nlm.nih.gov/geo/) database, The Cancer Genome Atlas (TCGA, https://portal.gdc.cancer.gov/) database and International Cancer Genome Consortium (ICGC, https://dcc.icgc.org/).

### 2.2 Processing of scRNA-seq data of the HCC **samples**

First, scRNA-seq data of 5,115 HCC cells of seven HCC samples were obtained from GSE125449 dataset set1 in the GEO database. Second, Seurat software package was used to perform quality control on the scRNA-seq data of the obtained HCC cells, and the HCC cells data were excluded that meet the following two criteria: genes detected in <3 cells were excluded, and cells with <200 total detected genes were excluded. Linear regression was used to standardize the remaining scRNA-seq data of HCC cells, and the hypervariable genes were identified through analysis of variance. Finally, under the condition that the cumulative contribution of the principal component (PC) was greater than 90%, the variance contribution of the PC itself was less than 5%, and the difference between two consecutive PCs was less than 0.1%, and the Principal Component Analysis (PCA) was used to screen out the dimensions with significant separation [12]. The t-Distributed Stochastic Neighbor Embedding (tSNE) and  Uniform Manifold Approximation and Projection algorithms were used for the dimensionality reduction of the first 15 PCs and for performing cluster analysis on HCC cells [13]. Cell populations were annotated based on marker genes through the “SingleR” package. According to the two standards of |log2 fold change (FC)| >0.5 and false discovery rate (FDR) <0.05, the marker genes in each cell population were obtained. The marker genes of HCC cells were obtained from the literature. Seurat’s visualization function was used to visualize gene expression across cell populations.

### 2.3 Trajectory analysis and HCC differentiation-related genes (HDRGs) identification

The pseudo-time and trajectory analysis of HCC cells through the “Monocle” package [14]. Then look for genes that were differentially expressed in different differentiation trajectories, the differentially expressed genes were selected unique to different differentiation trajectories and then were identified HDRGs, a total of 1,303 HDRGs. “clusterProfiler,” “org.Hs.eg.db,” “enrichplot,” and “ggplot2” packages were applied for Gene Ontology (GO) annotation and Kyoto Encyclopedia of Genes and Genomes (KEGG) enrichment analysis.

### 2.4 HDRG-based molecular classes of HCC patients from TCGA database

First, the “Sva” package and ComBat function were used to eliminate the batch effect between TCGA and ICGC; then 1,303 HDRGs are reserved for HCC molecular typing in the TCGA database. A “ConsensusClusterPlus” package that provides steady visual and quantitative evidence for measuring the number of unsupervised classes was used for HCC consensus clustering [15]. Partitioning around center was used to define the number of classes and carry out 50 iterations of max *K* = 9 for steady classes. Kaplan–Meier analysis was applied to estimate the survival situation, and the “ggplot2” package was applied to exhibit the proportion of clinicopathological features in each molecular class. In addition, the expression levels of HDRGs in specific molecular classes were studied in different cell differentiation trajectories, and they were represented by Heatmap.

### 2.5 Immune checkpoint genes (ICG) expression across molecular classes

About 41 proved ICGs were acquired from preceding research [2,16,17,18,19,20,21,22,23,24,25,26,27,28,29,30,31,32,33,34,35], and then, their expression across HCC classes was inspected by differential expression analysis through TCGA database. Kaplan–Meier analysis was applied to probe the prognostic worth of ICGs.

### 2.6 Generation, assessment, and confirmation of the HDRG-based prognostic risk score model

HDRGs were screened in TCGA cohort, and log2-scale conversion was used to standardize and correct the transcription profile. Then HDRGs were included in Weighted correlation network analysis (WGCNA), and key modules associated with HCC differentiation were determined through correlation analysis. Analyze genes in key modules through differential expression analysis (with |log2[FC]| > 1 and FDR < 0.05) and univariate analysis [*P* < 0.05]). Then, Bortuta was used to screen out 63 important genes, and these genes use the random forest to obtain 30 important genes and perform multivariate Cox regression analysis on the remaining HDRGs to generate HDRG-based prognostic RS model. The average value of HDRG-based prognostic RS model can be used to divide patients into high-risk groups and low-risk groups. RS can be calculated as the sum of the product of HDRG expression level and coefficient by the following formula:

\text{RS}=\mathop{\sum }\limits_{i}^{k}(\text{Exp}i\times \text{Coe}i),]

where ‘*i*’ represents the ‘*i*’th gene, and ‘*k*’ represents the total number of genes. TCGA and ICGC cohorts were used as the training cohort and the validating cohort, respectively. Kaplan–Meier analysis was used for the analysis of prognosis (http://www.oncolnc.org/); ROC curve was used to estimate accuracy.

### 2.7 Potential molecular-targeted drugs screening of RS-HDRGs

Based on the RNAactDrug database (http://bio-bigdata.hrbmu.edu.cn/RNAactDrug/), which includes an integrated analysis of three large pharmacogenomic databases (Genomics of Drug Sensitivity in Cancer, CellMiner, and Cancer Cell Line Encyclopedia), providing drug sensitivity and RNA molecules (including mRNAs, long non-coding RNAs, and microRNAs) at four molecular levels (expression, copy number variation, mutation, and methylation) association data.

### 2.8 Statistical analysis

All data in this study were statistically analyzed using R and Perl. The normality of the measurement data was first tested, and the measurement data that obeyed the normal distribution were expressed as mean ± standard deviation, and the comparison between groups was performed by *T*-test or analysis of variance. Enumeration data were expressed as numbers or frequencies (%), and comparisons between groups were performed using the *χ* ^2^ test. Survival analysis was performed using Kaplan–Meier analysis and log-rank test. *P* < 0.05 (two-sided) indicated a statistically significant difference.

## 3 Results

### 3.1 Identification and annotation of 16 cell clusters in human HCC samples based on scRNA-seq data

**Figure 1:**
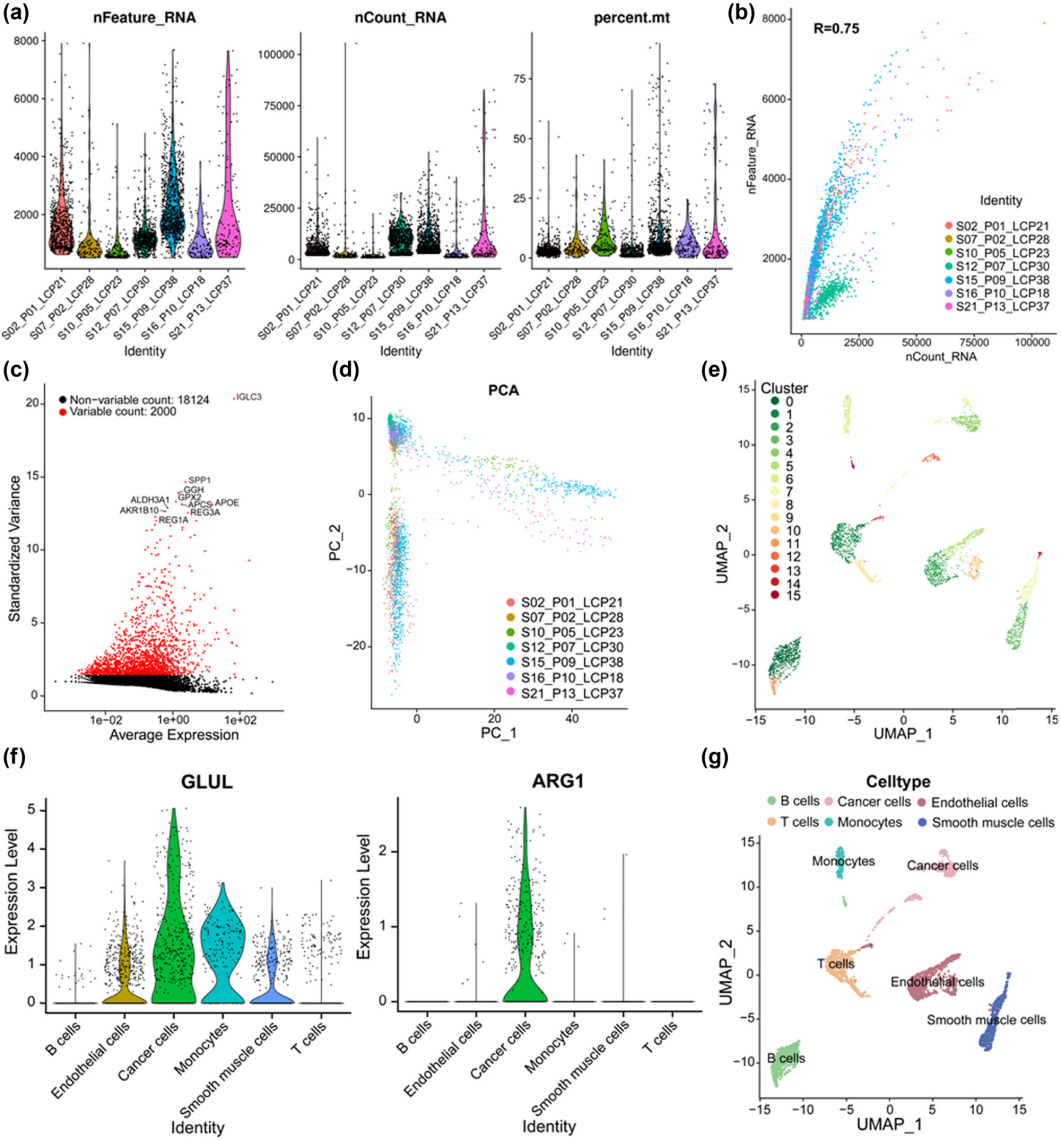
Identification and annotation of 16 cell clusters in human HCC samples based on scRNA-seq data. (a) According to quality control and normalization, 2,029 noncompliant cells were precluded, and 3,086 cells were involved in the analysis. (b) Correlation analysis between total intracellular gene sequences and sequencing depth. (c) A total of 20,124 genes were analyzed, of which 18,124 had low intercellular variation and 2,000 had high variation. (d) PCA. (e) 3,086 HCC cells were divided into 16 clusters. (f) Expression of HCC-related genes in 16 clusters. (g) 16 clusters were annotated. HCC: hepatocellular carcinoma, PCA: principal component analysis, PCs: principal components.

To begin, 5,115 cells were obtained from seven HCC samples via GSE125449. After undergoing quality control and normalization, 2,029 noncompliant cells were removed, leaving a total of 3,086 cells included in the analysis (Figure 1a). A significant correlation was observed between total intracellular gene sequences and sequencing depth (Figure 1b, *R* = 0.75). A total of 20,124 genes were analyzed across all cells from the HCC samples, with 18,124 genes exhibiting low intercellular variation and 20,00 genes with high intercellular variation (Figure 1c). PCA revealed that there was no significant separation among the HCC cells (Figure 1d). Utilizing the tSNE algorithm, the 3,086 HCC cells were divided into 16 clusters (Figure 1e). Among them, clusters 4, 8, and 12 have high expression of hepatocellular carcinoma-related genes [36], suggesting that these clusters were cancer cells (Figure 1f). Based on marker genes, each of the 16 clusters was annotated as follows: clusters 0, 11, and 15 were identified as B cells, cluster 1 and 9 as T cells, clusters 2, 5, 10, and 13 as endothelial cells, clusters 3, 7, and 14 as smooth muscle cells, clusters 4, 8, and 12 as cancer cells, and cluster 6 as monocytes (Figure 1g).

### 3.2 Identification of three states based on cell trajectory analysis and molecular functional analysis of three states based on HDRGs

**Figure 2:**
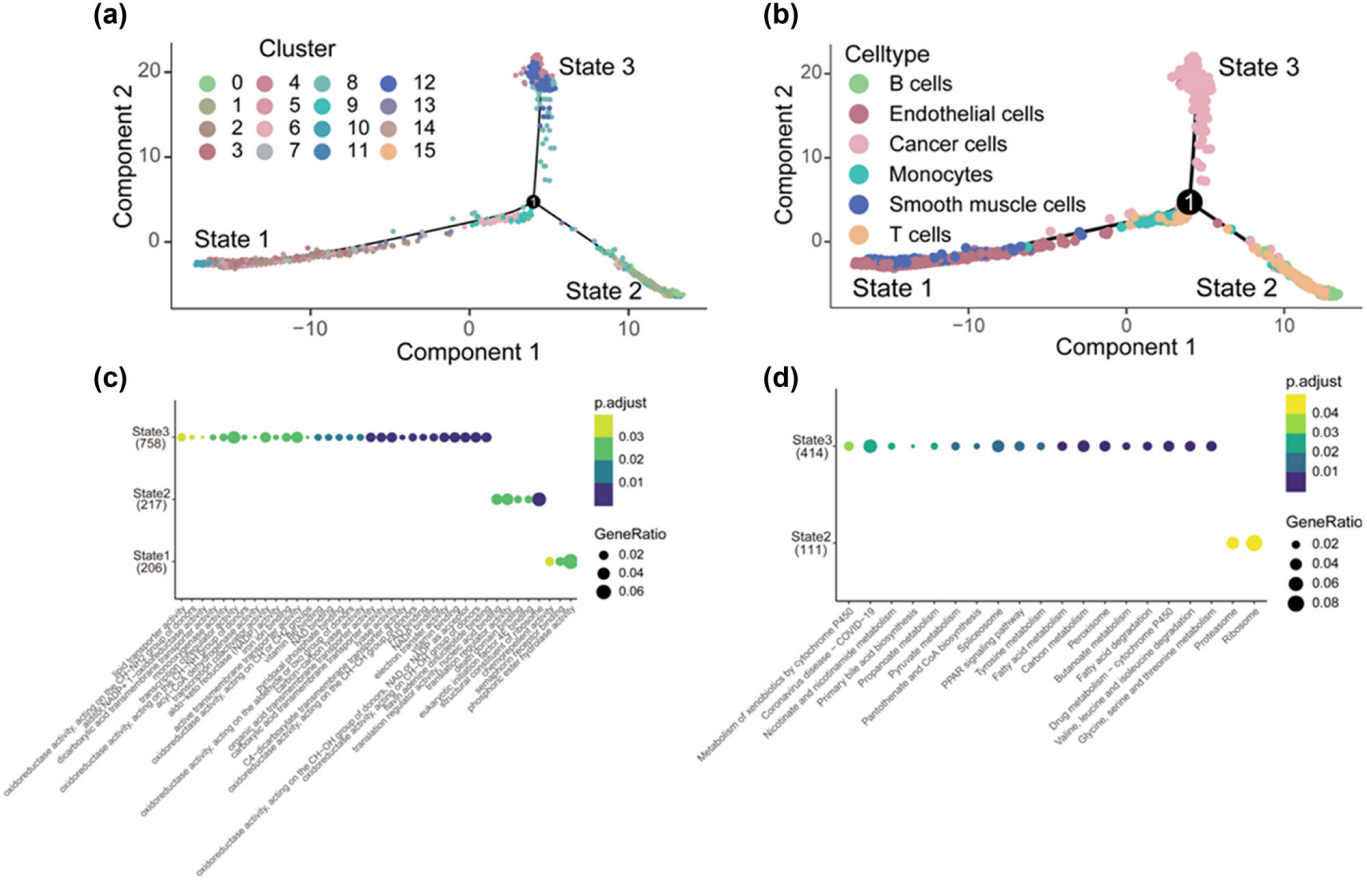
Identification of three states based on cell trajectory analysis and molecular functional analysis of three states based on HDRGs. (a) and (b) Pseudo-time and trajectory analysis. (c) GO analysis of State 1, State 2, and State 3 genes. (d) KEGG Pathway analysis of State 2 and State 3 genes. HDRGs: HCC differentiation-related genes, GO: Gene Ontology, KEGG: Kyoto Encyclopedia of Genes and Genomes.

Cell trajectory analysis demonstrated that all cells from the HCC samples were distributed across three states: State 1, primarily comprised of clusters 2, 3, 5, 6, 7, 10, 13, and 14; State 2, primarily comprising clusters 0, 1, 9, 11, and 15; and State 3, primarily comprising clusters 4, 8, and 12. Smooth muscle cells, monocytes, and endothelial cells were primarily distributed in State 1, while the majority of B cells and T cells were distributed in State 2, and cancer cells were primarily distributed in State 3 (Figure 2a and b). According to GO and KEGG pathway analysis, the highly differentially expressed genes (HDRGs) in State 1 were strongly associated with phosphoric ester hydrolase activity (Figure 2c); those in State 2 were involved in eukaryotic initiation factor 4E binding, translation regulator activity, and ribosome-related functions (Figure 2c and d); and those in State 3 were associated with oxidoreductase activity and metabolic processes (Figure 2c and d).

### 3.3 Identification and validation of the HDRG-based classification of HCC patients from TCGA database

**Figure 3:**
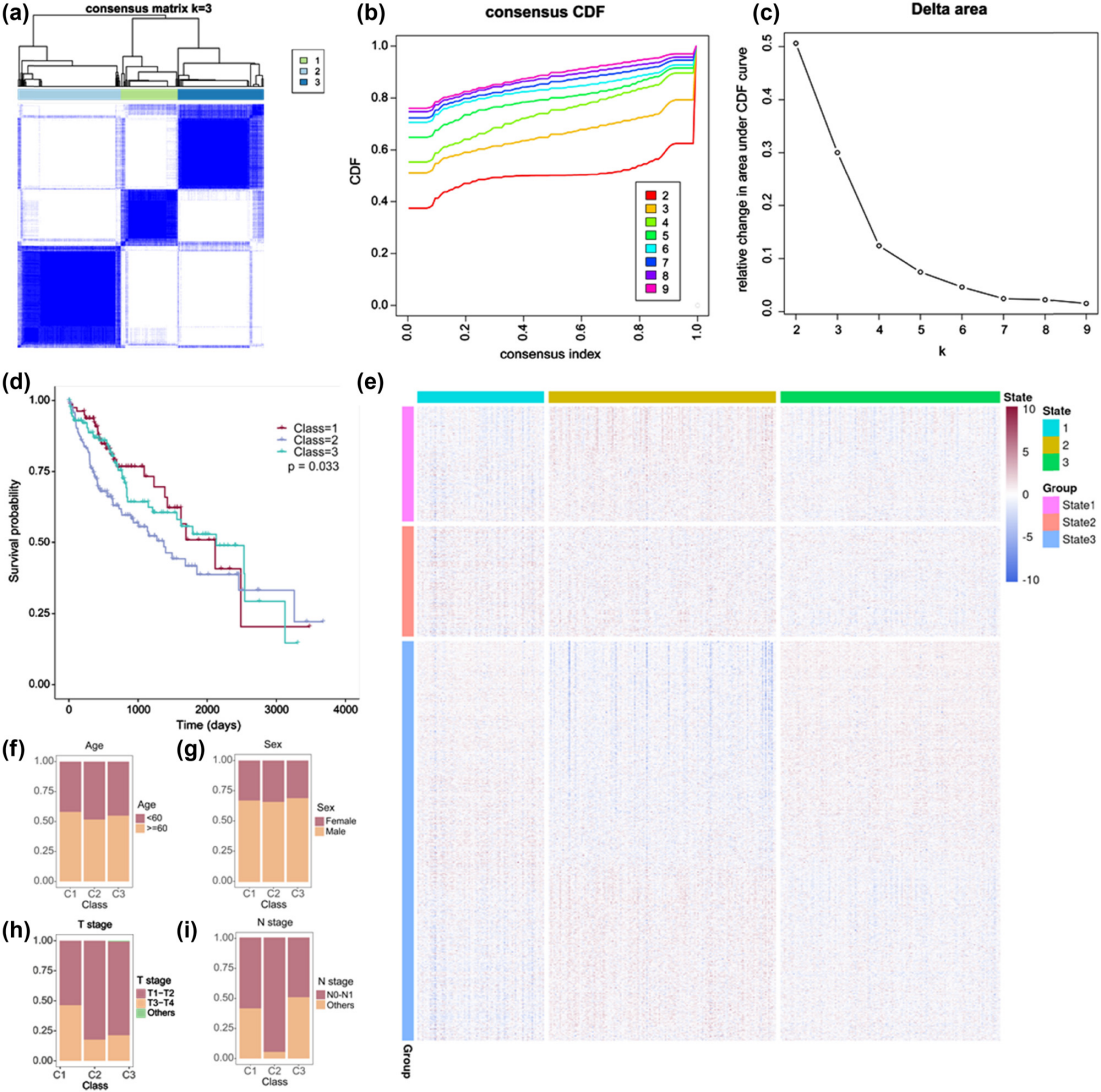
HDRG-based consensus clustering analysis of HCC patients from the TCGA database. (a–c) Three molecular classes were identified at a clustering threshold of *K* = 9. (d) Kaplan–Meier analysis among the three molecular classes. (e) The HDRGs in States 1/2/3 indicated the same cluster trends as Class 1/2/3. (f–i) Proportion of clinicopathologic features among the three molecular classes.

HDRG-based consensus clustering analysis was achieved in the TCGA database, and three molecular classes that included all the HCC samples were identified at a clustering threshold of max *K* = 9 (Figure 3a–c). Kaplan–Meier analysis showed that Class 2 (C2) patients had poorer overall survival (OS) than Class 1 (C1) patients and Class 3 (C3) patients, while compared with C3 patients, C1 patients had better 5-year OS and worse 10-year OS.

Kaplan–Meier analysis revealed that C2 patients had poorer OS compared to both C1 patients and C3 patients. Interestingly, C1 patients had better 5-year OS but worse 10-year OS compared to C3 patients (*P* = 0.033, Figure 3d). Furthermore, the HDRGs in State 1/2/3 showed the same cluster trend in Class 1/2/3 (Figure 3e), which suggested that Class 1/2/3 was individually composed of State 1/2/3. As C2 progressed to C3 and then C1, patient age increased (Figure 3f). There was no significant difference in the sex distribution among the three classes (Figure 3g). C2 patients tended to have early T stage and N stage (Figure 3h and i).

### 3.4 Expression levels of 41 ICGs across three molecular classes and prognostic analysis

**Figure 4:**
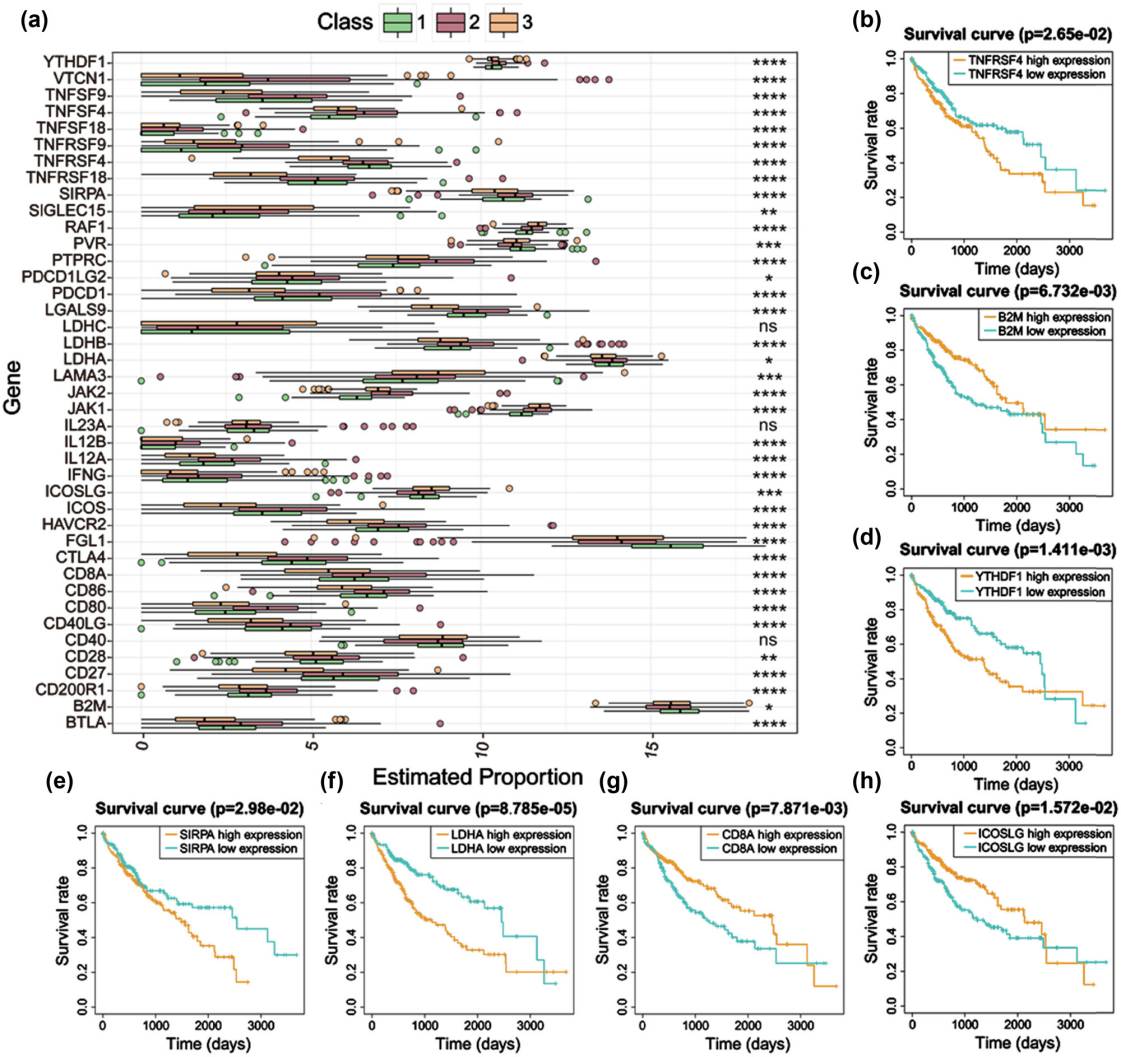
Expression levels of 41 ICGs across three molecular classes and prognostic analysis. (a) Differential expression analysis of 41 ICGs. (b–h) Kaplan–Meier analysis of TNFRSF4, B2M, YTHDF1, SIRPA, LDHA, CD8A, and ICOSLG.

About 41 proved ICGs were acquired from published research [2,16,17,18,19,20,21,22,23,24,25,26,27,28,29,30,31,32,33,34,35]. Differential expression analysis showed remarkably high expression of four ICGs (namely, TNFRSF4, PVR, FGL1, and B2M) in C1 (Figure 4a, all *P* < 0.05). Kaplan–Meier analysis revealed that high expressed TNFRSF4 predicted poor OS (overall survival) (Figure 4b, *P* < 0.05), while high expressed B2M predicted better OS (Figure 4c, *P* < 0.05). In C2, 30 ICGs (namely, YTHDF1, VTCN1, TNFSF9, TNFSF4, TNFSF18, TNFRSF9, TNFRSF18, SIRPA, PTPRC, PDCD1LG2, PDCD1, LGALS9, LDHB, LDHA, JAK1, JAK2, IL12B, IL12A, IFNG, ICOS, HAVCR2, cytotoxic T lymphocyte-associated protein-4 (CTLA4), CD8A, CD86, CD80, CD40LG, CD28, CD27, CD200R1, and BTLA) were significantly high expressed (Figure 4a, all *P* < 0.05), and Kaplan–Meier analysis demonstrated that high expressed YTHDF1, SIRPA, and LDHA predicted poor OS (Figure 4d–f, all *P* < 0.05), while high expressed CD8A predicted better OS (Figure 4g, *P* < 0.05). Four ICGs (namely, SIGLEC15, RAF1, LAMA3, and ICOSLG) were considerably high expressed in C3 (Figure 4a, *P* < 0.05), and Kaplan–Meier analysis indicated that high expressed ICOSLG predicted better OS (Figure 4h, *P* < 0.05). The above results showed that different ICGs have varying effects on OS in the three classes, which provides a rational molecular basis for guiding immunotherapy.

### 3.5 Generation, assessment, and confirmation of the HDRG-based prognostic risk score model

**Figure 5:**
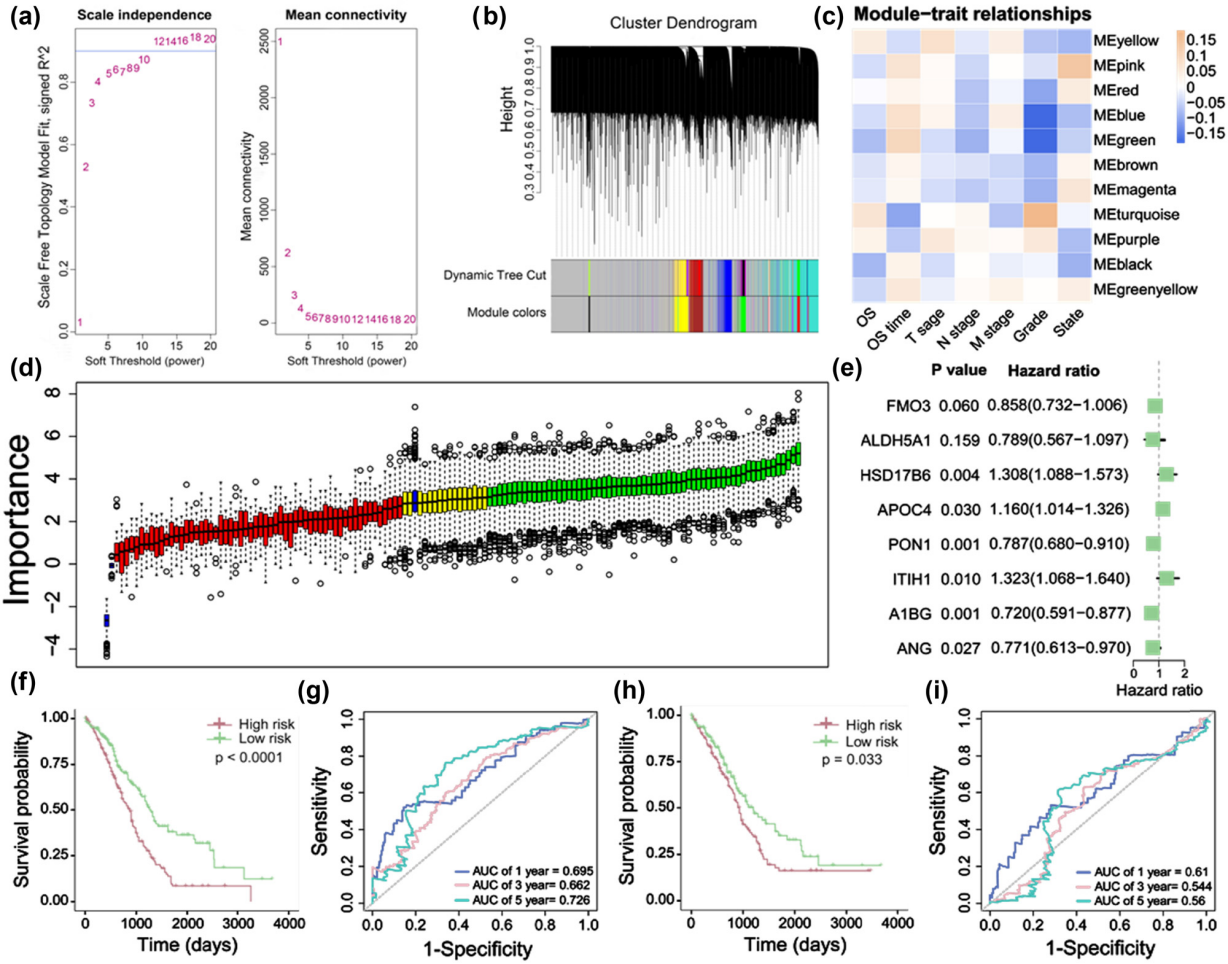
Generation, assessment, and confirmation of the HDRG-based prognostic risk score model. (a–b) Based on weighted correlation network analysis, 11 modules were accessed with a soft threshold = 10. (c) Correlation analysis between modules and clinicopathological data. (d) Variable filtering using Boruta, red represents low related genes, yellow represents uncertain genes and green represents high related genes. (e) Multivariate Cox regression analysis of differentially expressed HDRGs. (f) Kaplan–Meier analysis between the low-risk group and the high-risk group in the training cohort. (g) In the training cohort, the areas under the ROC curves for predicting 1-year, 3-year and 5-year OS. (h) Kaplan–Meier analysis between the low-risk group and the high-risk group in the validating cohort. (i) In the validating cohort, the areas under the ROC curves for predicting 1-year, 3-year, and 5-year OS.

A total of 1,303 HDRGs were obtained from the TCGA database and used for WGCNA. 11 Modules were accessed with soft threshold = 10 (Figure 5a and b), two modules of which (pink module and turquoise module) were positively associated with State and Grade of HCC patients (Figure 5c). A total of 137 differentially expressed HDRGs were identified from these two modules (Figure 5c), Then, using Boruta, 63 prognostic HDRGs were further screened (Figure 5d), and 30 important HDRGs were obtained from these genes by random forest (Figure S1). Finally, eight HDRGs were selected using multivariate Cox regression analysis to construct a prognostic risk scoring (RS) model. The RS of each sample could be calculated according to the relative coefficient and gene expression. RS = (−0.15258 × expression of FMO3) + (−0.23713 × expression of ALDH5A1) + (0.26864 × expression of HSD17B6) + (0.14836 × expression of APOC4) + (−0.23968 × expression of PON1) + (0.28024 × expression of ITIH1) + (−0.32831 × expression of A1BG) + (−0.25993 × expression of ANG) (Figure 5e). Based on the prognostic RS model, the RS of each HCC sample in the training and test cohorts was calculated, and it was found that the OS of the low-risk group was significantly better than that of the high-risk group (Figure 5f, training cohort: *P* < 0.0001; Figure 5h, test cohort: *P* < 0.05). Furthermore, the areas under the receiver operating characteristic (ROC) curves for predicting 1-year, 3-year and 5-year OS were 0.702, 0.646, and 0.656, respectively, in the training cohort (Figure 5g), and 0.61, 0.544, and 0.56, respectively, in the test cohort (Figure 5i). The clinicopathological characteristics of HCC patients in the TCGA training cohort and ICGC validating cohorts are summarized (Table 1).

**Table 1:** Clinicopathological characteristics of patients with HCC

Variable	TCGA Training cohort (*n* = 371)	ICGC Validating cohort (*n* = 228)
	*n* (%)	*n* (%)
Age (years)		
≥60	201 (54.18)	184 (80.70)
<60	170 (45.82)	44 (19.30)
Sex		
Male	250 (67.39)	168 (73.68)
Female	121 (32.61)	60 (26.32)
Stage		
I	−	36 (15.79)
II	−	103 (45.18)
III	−	70 (30.70)
IV	−	19 (8.33)
T stage		
T1	181 (48.79)	−
T2	94 (25.34)	−
T3	80 (21.56)	−
T4	13 (3.50)	−
TX	1 (0.27)	−
Unknown	2 (0.54)	−
N stage		
N0	252 (67.92)	−
N1	4 (1.08)	−
NX	114 (30.73)	−
Unknown	1 (0.27)	−
M stage		
M0	266 (71.70)	−
M1	4 (1.08)	−
MX	101 (27.22)	−

### 3.6 Expression, validation, and prognosis of eight RS-HDRGs

**Figure 6:**
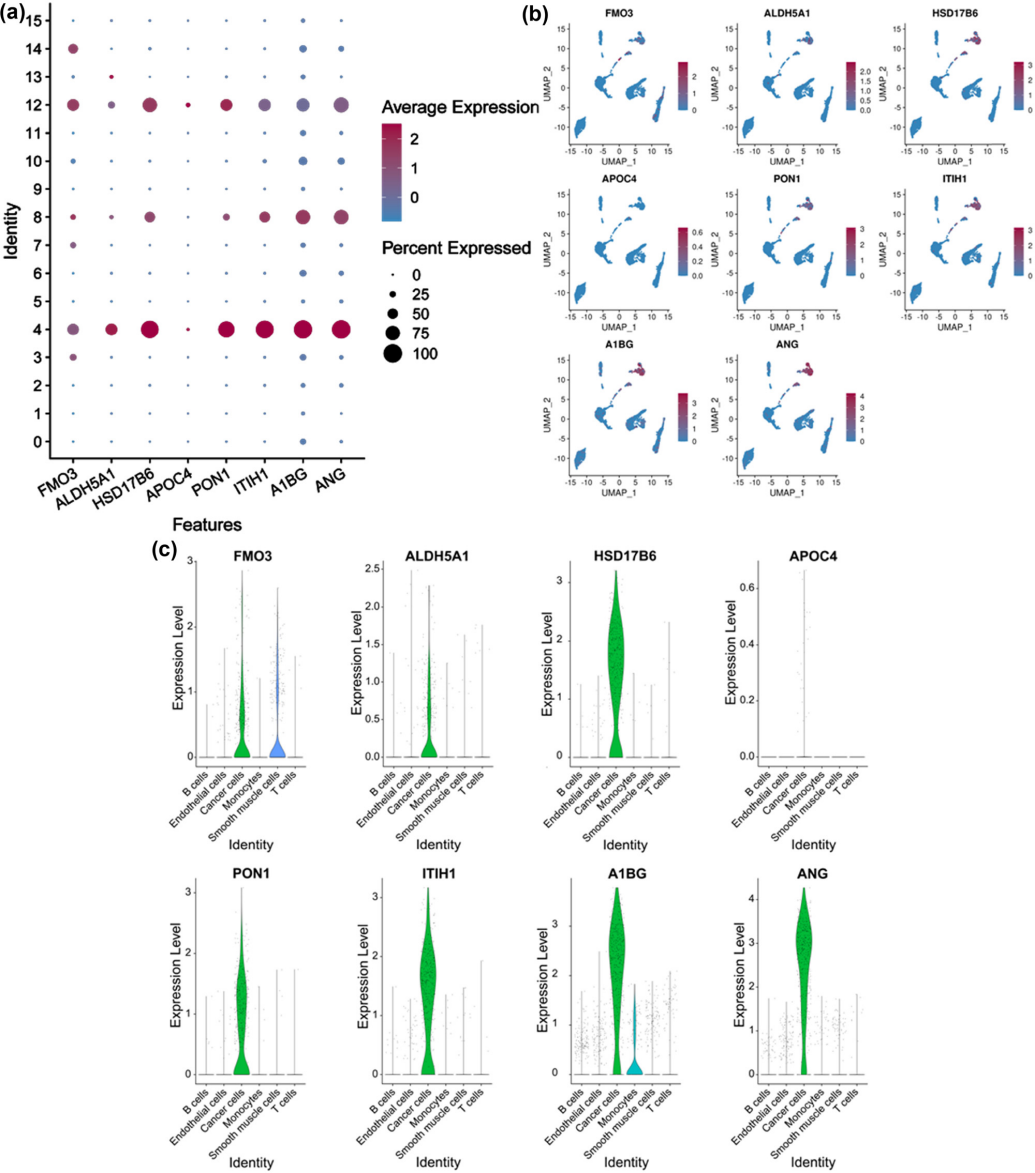
Expression and validation of eight RS-HDRGs. (a) and (b) Expression levels of eight RS-HDRGs in 16 clusters. (c) Expression levels of eight RS-HDRGs in all cells from seven HCC samples.

**Figure 7:**
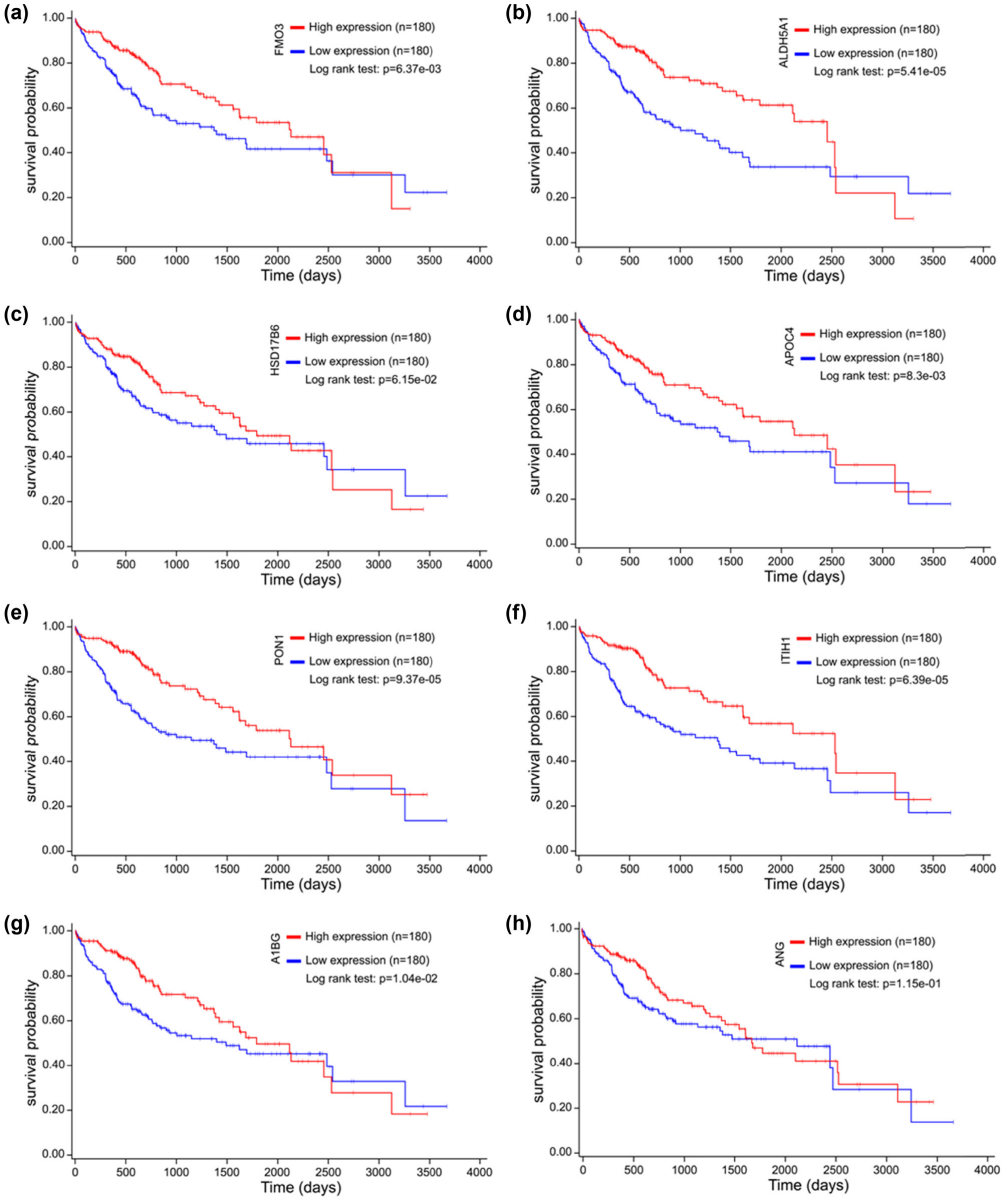
Prognostic analysis of eight RS-HDRGs based on TCGA database. (a–h) Kaplan–Meier analysis of FMO3, ALDH5A1, HSD17B6, APOC4, PON1, ITIH1, A1BG, and ANG.

The expression levels of the eight RS-HDRGs across 16 clusters are shown in the figure below, with the high expression levels observed in clusters 4, 8, and 12 (most of the cells in clusters 4/8/12 belonging to State 3) (Figure 6a and b). The expression patterns of these eight RS-HDRGs were found to be consistent with the above-mentioned marker genes of HCC cells, and they were all highly expressed in HCC cells. These findings suggest that these eight RS-HDRGs have the potential to serve as biomarkers of HCC cells (Figure 6c). Kaplan–Meier analysis of these eight RS**-**HDRGs indicated that high expression of FMO3, ALDH5A1, HSD17B6, APOC4, PON1, ITIH1, A1BG, and ANG were associated with better OS (Figure 7a–h).

### 3.7 Potential therapeutic drugs analysis of seven RS-HDRGs

**Figure 8:**
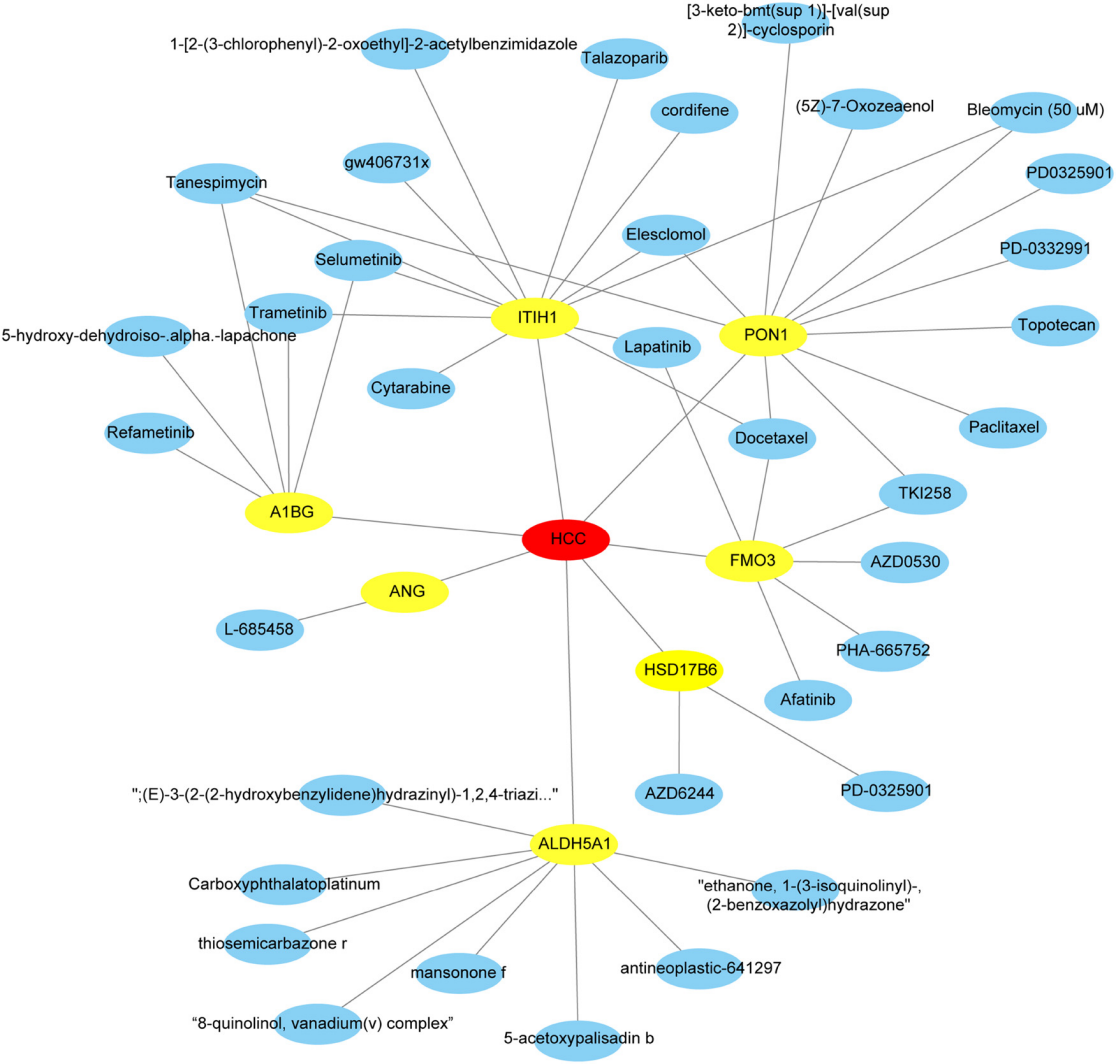
Compound-target network of seven RS-HDRGs of HCC. The yellow ones represent the seven RS-HDRGs, respectively. The blue ones represent molecularly targeted drugs, respectively. HCC: hepatocellular carcinoma.

This study screened potential therapeutic drugs for seven of the RS-HDRGs based on RNAactDrug database. The results showed that FMO3 was associated with drugs such as Lapatinib, Dovitinib (TKI258), and Docetaxel. ALDH5A1 was associated with drugs such as Carboxyphthalatoplatinum and Mansonone F. HSD17B6 was associated with drugs such as Selumetinib (AZD6244) and PD-0325901. PON1 was associated with drugs such as Dovitinib (TKI258), Docetaxel, and PD-0325901. ITIH1 was associated with drugs such as Lapatinib, Trametinib, and Selumetinib. A1BG was associated with drugs such as Refametinib, Trametinib, and Selumetinib. ANG was associated with L-685458 (Figure 8).

## 4 Discussion

In recent years, researchers have increasingly recognized that ITH in HCC is closely linked to disease progression, classification, treatment, and prognosis [37]. While CSCs are considered to be the main drivers of ITH, some studies have investigated the differentiation of HCC CSCs [38,39]. However, there were very few studies on the differentiation status of HCC cells. However, few studies have explored the differentiation status of HCC cells and their relationship with immunotherapy, molecular-targeted drugs, and clinical prognosis. Therefore, in this study, we further explored the heterogeneity of HCC cells by analyzing their differentiation trajectory. Our research results revealed that HCC cells could be classified into three distinct differentiation states, and we identified important HDRGs associated with cell subgroups. Importantly, the classification of HCC patients based on the differentiation state of HCC cells played a crucial role in immunotherapy, identifying potential molecularly targeted drugs, and clinical prognosis for HCC patients.

The scRNA-Seq technology provides a unique perspective for studying ITH [40]. ITH means that within a tumor, all tumor cells do not have a single set of tumor molecular characteristics, but have rather their own relatively specific molecular characteristics or differentiation status [41]. In this study, we identified 16 cell clusters in the scRNA-Seq data obtained from seven HCC samples, which were consistent with previous works of literature [42]. Using Gene Set Enrichment Analysis and correlation analysis, we found that genes associated with State 1 were closely related to phosphoric ester hydrolase activity, genes associated with State 2 were involved in eukaryotic initiation factor 4E binding, translation regulator activity, and ribosomes, and genes associated with State 3 were involved in oxidoreductase activity and metabolism. These findings suggest that the differentiation status of HCC cells can reveal the intratumoral heterogeneity of HCC and is related to intratumoral metabolism pathways. This information may provide a reference basis for individualized treatment of HCC patients.

In recent years, immunotherapy has emerged as a promising treatment for tumors. By blocking the immune escape mechanisms of tumors and restoring the killing functions of immune cells, it offers new possibilities for the treatment and even cure of cancers [43]. Studies have shown that immune checkpoint inhibitors, such as programmed death-1 antibodies, programmed death ligand-1 (PD-L1) antibodies, and (CTLA-4) antibodies, have demonstrated certain efficacy in the clinical treatment of HCC [4,44,45]. Clinical studies on immunotherapy for HCC, from second-line to first-line therapy, and perioperative therapy are also underway, with promising prospects for the future of HCC immunotherapy. However, the clinical guidance value of PD-L1, which is a widely used biomarker for different tumors, is not uniform. Studies have shown that the positive expression rate of PD-L1 in HCC tumor cells is no more than 10% [46]. Therefore, it is important to search for effective predictive markers for immunotherapy of tumor. In this study, we found that HCC could be divided into three classes based on HDRGs, and we observed a correlation between the different classes and OS as well as the expression of ICGs. These relationships are similar to previous studies [47]. Thus, based on the above findings, we believed that the characteristics of HCC cell differentiation status can serve as an effective predictor of HCC immunotherapy. Immune checkpoint inhibitors have the potential to break through the bottleneck of advanced HCC treatment and improve clinical efficacy by screening for the dominant population and optimizing drug combinations in the future. This area is worthy of further research and exploration.

Studies also reported that patients’ classifications through tumor differentiation-related genes can be used to predict OS in tumor patients. Several studies have reported that patient classifications based on tumor differentiation-related genes can be used to predict OS in cancer patients [47,48]. In this study, we identified eight RS HDRGs (namely, FMO3, ALDH5A1, HSD17B6, APOC4, PON1, ITIH1, A1BG, and ANG) and explored the impact of HDRGs on OS of patients with HCC. Survival analysis showed that patients with C2 HCC had a poor prognosis, while the survival rates of patients with C1 HCC and C3 HCC were better, indicating that patient classifications based on HDRGs can be used to predict the survival of HCC patients. Based on the above HDRGs, we constructed a risk-scoring model for predicting the survival of HCC patients. This RS model can help clinicians to predict the survival of HCC patients and provide new insights for clinical decision-makings. Flavin-containing monooxygenase 3 (FMO3) belongs to the flavin oxidase family and is an important liver microsomal enzyme involved in the oxidative metabolism of drugs, xenobiotics, and other chemicals in the body. One study showed that ectopic re-expression of FMO3 increased apoptosis and decreased cell viability in liver-derived cancer cell lines [49]. Its family member, FMO4, also represented a worse prognosis in HCC [50]. Aldehyde dehydrogenase 5 family member A1 (ALDH5A1) encodes succinate semialdehyde dehydrogenase, which is an enzyme involved in mitochondrial glutamate metabolism. ALDH5A1 is positively associated with prognosis in patients with ovarian cancer, particularly in those with serous ovarian cancer [51]. Hydroxysteroid 17-Beta Dehydrogenase 6 (HSD17B6) encodes a protein with both oxidoreductase and isonomerase activities involved in steroid metabolism. HSD17B6 expression is lower in HCC than in normal liver tissue, and it can inhibit the proliferation, migration, and invasion of HCC cells, and is related to tumor grading and staging. Low expression of HSD17B6 in HCC patients is associated with poorer OS [52,53]. And high expression of apolipoprotein C4 (APOC4) in HCC patients is associated with better OS [54]. Paraoxonase 1 (PON1), a member of the paraoxonase family, which encodes an enzymatic protein with lactonase and esterase activities. Down-regulation of PON1 may suggest that patients with liver cancer have difficulty in survival [55]. PON1 in serum can be used as a biomarker for early diagnosis of HCC and for the status of vascular invasion[56,57]. Compared with the corresponding normal tissues, the expression of Inter-alpha-trypsin inhibitor heavy chain 1 (ITIH1) is significantly reduced in HCC, and its downregulation adversely affects patients’ prognosis [58]. In this study, we identified eight RS HDRGs that were associated with better OS in HCC patients when they were highly expressed in HCC cells, and our findings were consistent with the earlier findings.

Most HCC patients are diagnosed in the middle or advanced stages when their tumors were found, which often precludes surgical resection as a treatment option. However, the combinations of molecularly targeted and immunotherapy can downstage advanced HCC and obtained the opportunity for surgery. Therefore, it is necessary to explore molecularly targeted drugs for patients with advanced HCC. In this study, we used the RNAactDrug database to identify drugs that may inhibit tumor progression by acting on RS-HDRGs, which could potentially improve the survival and prognosis of HCC patients. Based on these findings, we propose that the characteristics of HCC cell differentiation status can be used as an effective predictor of potential therapeutic drugs for HCC. This potential approach to improve the efficacy of HCC drug treatments is a current one among all feasible solutions. All these results were not found in previous studies.

One limitation of this study is that it is a retrospective analysis based on data from public databases. Therefore, the validity and practicality of our conclusions still require verification through further analysis of real-world data. In addition, while we identified potential therapeutic drugs for HCC based on our analysis of RS-HDRGs, further experimental studies are needed to validate their efficacy and safety.

## 5 Conclusions

This study uses bioinformatics algorithms to classify HCC cells into three subgroups based on the molecular typing of HDRGs, which can predict the immunotherapy response and prognosis of HCC patients and screen out potential molecular-targeted drugs. These findings provide important guidance for identifying subgroups of HCC tumors that may be susceptible to immunotherapy or potential molecular-targeted drugs. This will open a new perspective for individualized treatment strategies for HCC.

## Supplementary Material

Supplementary material
