# Supplementary material for: Hepatocellular carcinoma cell differentiation trajectory predicts immunotherapy, potential therapeutic drugs, and prognosis of patients
Source: Open Life Sci. 2023 Aug 8;18(1):20220656. doi: 10.1515/biol-2022-0656 (PMC10426728; doi:10.1515/biol-2022-0656)
Supplement: Supplementary material [file biol-2022-0656-sm.pdf]

Supplementary material

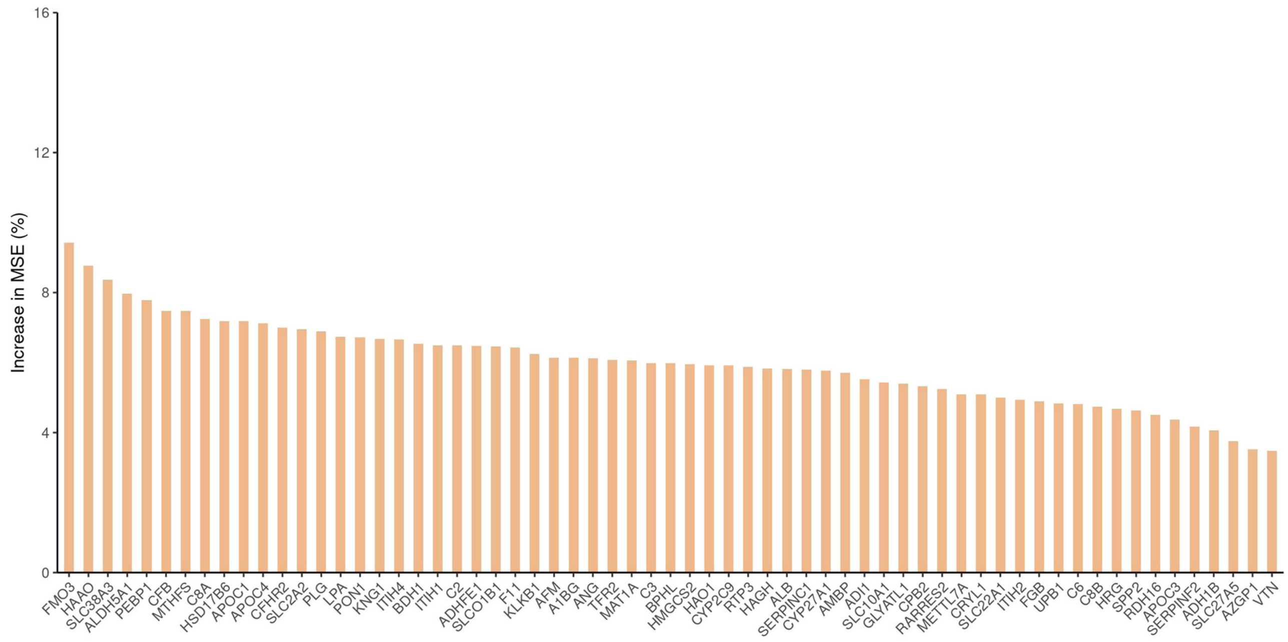

Figure S1: 30 important HDRGs were screened out by random forest algorithm.

Table S1: Correlation coefficients of drugs and genes

Compound	RNAtype	RNAmolecule	Omics	Source	Spearman.stat	Spearman.fdr
5-hydroxy-dehydroiso-.alpha.-lapachone	mRNA	A1BG	Expression	CellMiner	0.508331386212532	0.0182260203378419
Refametinib	mRNA	A1BG	Expression	GDSC	0.158796841518373	$3.89768473203669 \times 10^{-6}$
Trametinib	mRNA	A1BG	Expression	GDSC	0.154056809134226	$6.92507800635304 \times 10^{-6}$
Tanespimycin	mRNA	A1BG	Expression	GDSC	0.127373420647314	0.000224459239584767
Selumetinib	mRNA	A1BG	Expression	GDSC	0.101013427281606	0.00505290014564572
5-acetoxypalisadin b	mRNA	ALDH5A1	Expression	CellMiner	0.504430142849642	0.0374420873803966
ethanone, 1-(3-isoquinolinyl)-, (2-benzoxazolyl)hydrazone	mRNA	ALDH5A1	Expression	CellMiner	0.497132630678113	0.0307379051279078
thiosemicarbazone r	mRNA	ALDH5A1	Expression	CellMiner	0.445618281585896	0.0258373866341666
8-quinolinol, vanadium(v) complex	mRNA	ALDH5A1	Expression	CellMiner	0.425842307383906	0.0388144141581997
antineoplastic-641297	mRNA	ALDH5A1	Expression	CellMiner	0.413604346191958	0.039728063000351
;(E)-3-(2-(2-hydroxybenzylidene) hydrazinyl)-1,2,4-triazi...	mRNA	ALDH5A1	Expression	CellMiner	0.401150433309071	0.0338566955549805
Carboxyphthalatoplatinum	mRNA	ALDH5A1	Expression	CellMiner	0.386905840098404	0.0428833107137504
mansonone f	mRNA	ALDH5A1	Expression	CellMiner	0.371022870658699	0.0405295942817403
L-685458	mRNA	ANG	Expression	CCLE	0.134801175586109	0.0496138440962285
Lapatinib	mRNA	FMO3	Expression	CCLE	0.2493922087357	0.00168298536748495
AZD0530	mRNA	FMO3	Expression	CCLE	0.216831790316606	0.0226663724439086
PHA-665752	mRNA	FMO3	Expression	CCLE	0.153886348128145	0.0456279824768029
TKI258	mRNA	FMO3	Expression	CCLE	0.147618387353001	0.0367611362823351
Afatinib	mRNA	FMO3	Expression	GDSC	0.0932142381779823	0.0152365067821484
Docetaxel	mRNA	FMO3	Expression	GDSC	0.0760734348722215	0.0348859459404401
PD-0325901	mRNA	HSD17B6	Expression	CCLE	0.320772240046469	0.0000161393458646967
AZD6244	mRNA	HSD17B6	Expression	CCLE	0.277922839553241	0.000260172500363559
1-[2-(3-chlorophenyl)-2-oxoethyl]-2-acetylbenzimidazole	mRNA	ITIH1	Expression	CellMiner	0.453598078044486	0.0271310497859967
cordifene	mRNA	ITIH1	Expression	CellMiner	0.401192113748032	0.0483909951047506
gw406731x	mRNA	ITIH1	Expression	CellMiner	0.386426350004577	0.0497977937575667
Docetaxel	mRNA	ITIH1	Expression	GDSC	0.203263619722557	$4.59188475880994 \times 10^{-9}$
Bleomycin (50 μM)	mRNA	ITIH1	Expression	GDSC	0.178993880917601	$2.66716732044866 \times 10^{-7}$
Lapatinib	mRNA	ITIH1	Expression	CCLE	0.170443718553456	0.0414969457656631
Tanespimycin	mRNA	ITIH1	Expression	GDSC	0.111338592344489	0.00133682361532356
Talazoparib	mRNA	ITIH1	Expression	GDSC	0.0963333194652519	0.0210228194931732
Cytarabine	mRNA	ITIH1	Expression	GDSC	0.0858881622027268	0.0484188607087372
Elesclomol	mRNA	ITIH1	Expression	GDSC	0.082277160060289	0.0459365787498578
Selumetinib	mRNA	ITIH1	Expression	GDSC	0.0819819911604118	0.0250140061225786
Trametinib	mRNA	ITIH1	Expression	GDSC	0.0771895157838321	0.0301848634886606
[3-keto-bmt(sup 1)]-[val(sup 2)]-cyclosporin	mRNA	PON1	Expression	CellMiner	0.367588627196993	0.0469299733565379
PD-0332991	mRNA	PON1	Expression	CCLE	0.215988057259187	0.00118708164634315
TKI258	mRNA	PON1	Expression	CCLE	0.214376145187218	0.00165207237780736

(Continued)

Table S1: *Continued*

Compound	RNAtype	RNAmolecule	Omics	Source	Spearman.stat	Spearman.fdr
Paclitaxel	mRNA	PON1	Expression	CCLE	0.151848409973872	0.0465558144047938
Docetaxel	mRNA	PON1	Expression	GDSC	0.151372832185567	0.0000158424844158286
Topotecan	mRNA	PON1	Expression	CCLE	0.1510989317282	0.0278073194593523
Bleomycin (50 µM)	mRNA	PON1	Expression	GDSC	0.116483010393672	0.00101183292034756
Elesclomol	mRNA	PON1	Expression	GDSC	0.112304706805083	0.00454683350220454
PD0325901	mRNA	PON1	Expression	GDSC	0.0890258130810364	0.0131201943984483
(5Z)-7-Oxozeaenol	mRNA	PON1	Expression	GDSC	0.0804680921469839	0.0425157057127624
Tanespimycin	mRNA	PON1	Expression	GDSC	0.0790974417199523	0.0248663749011228
